# Medical Efficiency Pays

**Published:** 1917-04

**Authors:** 


### Medical Efficiency Pays.

Yesterday a patient, a young woman teacher, was bemoaning the fact that she failed to be promoted because for two years she has taken no educational journal or attended a summer course. Now she takes three journals and she goes to Columbia next summer. Another, a woman music teacher, resigned a good position because she had not been to the conservatory for post-graduate study for three years, and she "just must go or drift behind."

A few days ago we were called to another town in consultation. The prominent-citizen patient had consulted nearly every doctor in town, at 50 cents per. His family physician was angry because a "young ass just out of a hospital job had charged $5.00 and said all the rest were wrong." It transpired that the "young ass" was right in his conclusion, and he was the only doctor in the town who had made an adequate examination.

Yes, thousands of doctors simply don't count, and it's a pity, a great pity. A teacher is not necessarily a teacher, nor a musician a musician; neither is merely a doctor a *real doctor*.—*The Medical Council.*

---

The twentieth annual meeting of the Association of Medical Officers of the Army and Navy of the Confederate States will be held in the New Willard Hotel, headquarters of the United Confederate Veterans, Washington, D. C., June 4, 5, 6, 7, 8, 1917.

All those who were surgeons, assistant surgeons, or acting assistant surgeons and chaplains of the Confederate army or navy, and all those who served in the army or the navy as soldiers or sailors—not then medical officers—but who, after the war, became regular practitioners of medicine in good standing; and all regular practitioners of medicine whose fathers or grandfathers served in the Confederate army or navy are eligible to full membership.

And all those who served as matrons or nurses in the hospitals or in the field are welcomed to honorary membership.

The objects of the association are to collect all official records and important facts, as far as may be possible, relating to the history of the medical departments of the army and navy of the Confederate States; to ascertain the military records of all the officers and prepare a roster of the same; to honor the memory of its deceased members, and the memory of the nurses; and otherwise, not already mentioned, to perpetuate the history of said departments and of this association.

Further information will be supplied upon application to the secretary.

CARROLL KENDRICK, M. D., President,
Kendrick, Miss.

